# Complete Genome Sequence of Escherichia coli O157:H7 Phage PhiG17

**DOI:** 10.1128/MRA.01296-18

**Published:** 2019-01-17

**Authors:** Muyiwa A. Akindolire, Bukola R. Aremu, Collins N. Ateba

**Affiliations:** a Department of Microbiology, School of Biological Sciences, Faculty of Natural and Agricultural Sciences, North-West University, Mmabatho, South Africa; b Food Security and Safety Niche Area, North-West University, Mmabatho, South Africa; Queens College

## Abstract

Here, we announce the complete genome sequence of bacteriophage PhiG17, which is virulent to Escherichia coli O157:H7 strains and was isolated from cattle feces in the North West province of South Africa. This report presents the major genetic features of the phage PhiG17 based on its whole-genome sequence.

## ANNOUNCEMENT

**FIG 1:**
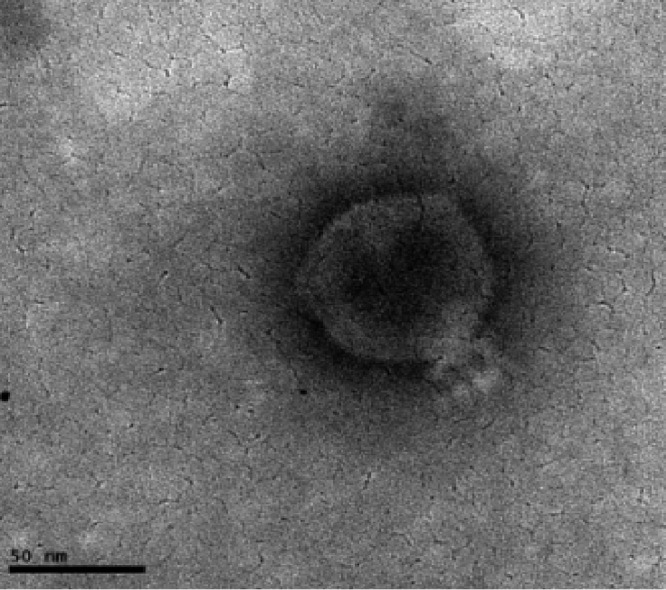
Electron micrograph of *E. coli* O157:H7 phage PhiG17. Bar = 50 nm.

Escherichia coli O157:H7 is an important foodborne pathogen of significant public health concern worldwide (1, 2). In order to prevent human exposure to this pathogen, virulent bacteriophages have been suggested as a promising tool that can be used in reducing E. coli O157:H7 in both farm animals and food products (3 – 7). In the application of these promising biocontrol and therapeutic agents, it is important to screen the genomes of potential candidates to ensure their safety and efficacy (8). In this report, we present for the first time the complete genome sequence of a PhiG17 phage isolated from cattle feces in South Africa. The phage was capable of infecting virulent environmental Shiga toxin-producing E. coli (STEC) O157:H7 strains that were characterized in a previous report (9). Transmission electron micrographs of phage particles revealed an icosahedral head (75 ± 3.6 nm) and a short tail (23 ± 3.1 nm), as shown in Fig. 1, which are typical of the *Podoviridae* family (10).

Phage G17 genomic DNA was extracted and purified using a Norgen phage DNA isolation kit (Norgen Biotek Corp., Ontario, Canada) according to the manufacturer’s instructions. Purified phage DNA was sequenced at Inqaba Biotechnical Industries (Pty) Ltd. in South Africa, where the phage DNA was fragmented using an ultrasonication approach (Covaris). DNA fragments were size-selected (300 to 800 bp) using AMPure XP beads, the fragments were end-repaired, and Illumina-specific adapter sequences were ligated to each fragment. The sample was indexed, and a second size selection was performed. The sample was then quantified using a fluorometric method, diluted to a standard concentration (4 nM), and then sequenced on an Illumina MiSeq v3 platform (600-cycle kit) following a standard protocol as described by the manufacturer. A total of 50 Mb of data (2 × 300-bp long paired-end reads) were produced for the sample, and a total of 154,330 reads with an average read length of 274.9 bp were obtained, filtered, and trimmed using Trimmomatic v0.36 with default parameters (11). Further quality checking was done with FastQC v0.11.5 (http://www.bioinformatics.babraham.ac.uk/projects/fastqc), and *de novo* assembly of raw data was carried out using SPAdes v3.11.1 genome assembler software; the result was evaluated with QUAST (12).

The complete genome sequence of phage G17 revealed a genome size of 68,270 bp assembled in a single contig with an *N*_50_ value of 68,270 bp and an average G+C content of 43.5%. Genes and coding sequences in the genome were predicted and annotated using the PHAge Search Tool (PHAST) (13) and Rapid Annotations using Subsystems Technology (RAST) v2.0 server (14) online analysis tools. Predicted proteins in the genome were further annotated by BLAST against the NCBI nonredundant GenBank database (15). The PhiG17 genome contained a total of 78 coding sequences ranging in length from 114 bp to 10,353 bp and 1 isoleucine-tRNA as predicted by ARAGORN (16) and tRNAscan-SE (17). The genome lacks genes encoding known antibiotic resistance as determined by ResFinder v3.0 (18). Genes encoding toxins and those implicated in transduction were absent from the genome of phage PhiG17. Nucleotide BLAST search of the genome revealed 94% nucleotide similarity with APEC7 (GenBank accession number KF562340) and 95% with APEC5 (GenBank accession number KF192075), both of which are members of the newly assigned *G7cvirus*. Further CoreGenes analysis (18) of the genome revealed a significant similarity to the same recently assigned *G7cvirus*, notably *Escherichia* phage vB EcoP PhAPEC7 (GenBank accession number KF562340). Members of this recent group are classified as belonging to the *Podoviridae* family and the *G7cvirus* genus and are considered to be safe biocontrol candidates (19, 20). Thus, *Escherichia* phage G17 is suggested to be a member of the G7Cviruses.

### Data availability.

The genome sequence of *Escherichia* phage PhiG17 has been deposited in the NCBI GenBank database under the accession number MH358458. The raw sequences are available in the NCBI SRA database under the accession number SRP159624.
